# Frequency and Clinical Manifestations of Dengue in Urban Medellin, Colombia

**DOI:** 10.1155/2014/872608

**Published:** 2014-05-29

**Authors:** Berta Nelly Restrepo, Mark E. Beatty, Yenny Goez, Ruth E. Ramirez, G. William Letson, Francisco J. Diaz, Leidy Diana Piedrahita, Jorge E. Osorio

**Affiliations:** ^1^Instituto Colombiano de Medicina Tropical, Universidad CES, Sabaneta, Antioquia, Colombia; ^2^Biogen Idec, Cambridge, MA 02142, USA; ^3^El Paso County Public Health, Colorado Springs, CO 80907, USA; ^4^Grupo Inmunovirología, Facultad de Medicina, Universidad de Antioquia, Medellín, Colombia; ^5^Department of Pathobiological Sciences, School of Veterinary Medicine, University of Wisconsin, Madison, WI 53706, USA

## Abstract

A dengue fever surveillance study was conducted at three medical facilities located in the low-income district of San Javier in Medellin, Colombia. During March 2008 to 2009, 781 patients with fever regardless of chief complaint were recruited for acute dengue virus infection testing. Of the 781 tested, 73 (9.3%) were positive for dengue infection. Serotypes DENV-2 (77%) and -3 (23%) were detected by PCR. One patient met the diagnostic criteria for dengue hemorrhagic fever. Only 3 out of 73 (4.1%) febrile subjects testing positive for dengue infection were diagnosed with dengue fever by the treating physician. This study confirms dengue virus as an important cause of acute febrile illness in Medellin, Colombia, but it is difficult to diagnose without dengue diagnostic testing.

## 1. Introduction

Dengue virus is the arbovirus that causes dengue fever (DF). DF is a growing public health concern in most tropical countries. Because its primary vector,* Aedes aegypti*, prefers breeding in artificial containers commonly found in peridomestic areas; the burden of DF will continue to increase with the population of tropical cities. Currently, dengue causes about 100 million symptomatic cases and 25,000 deaths annually [1]. Infection can be asymptomatic or cause a range of severity from mild DF to dengue hemorrhagic fever (DHF) which can then progress to dengue shock syndrome (DSS) and death [2]. The dengue virus complex contains four antigenically and genetically distinct serotypes (DENV-1, -2, -3, and -4).

In Colombia, all four dengue serotypes are actively circulating. During the last 10 years there has been a significant increase in the number of cases of DF/DHF. In 1998, about 58,000 cases were reported, which increased to 157,152 cases in 2010 [3]. Medellin, the capital of Antioquia province, is the second largest city in the country, and its metropolitan area is home to 3,000,000 inhabitants. In Colombia, dengue virus transmission occurs year-round in a seasonal pattern with periodic epidemics resulting in several fold-higher transmission. Reported DF in Medellin climbed from 341 cases in 2009 to 17,456 in 2010, reflecting an increased incidence from 17.2 to 745.4 per 100,000 inhabitants, respectively (http://www.dssa.gov.co/).

While surveillance programs have been established to track epidemics and circulating strains, the true disease burden is underestimated due the focus on hospitalized DHF cases in most national surveillance systems [4]. Milder forms of dengue infection, which represent the largest proportion of cases, may also be misdiagnosed by treating physicians. The reasons for this include the lack of pathognomonic symptoms and access to appropriate cost effective dengue diagnostics.

We conducted the current study to characterize the frequency of symptomatic dengue infection, particularly the milder forms, in Medellin and confirm the suspicion of under reporting.

## 2. Method

### 2.1. Geographic Location

Medellin is located in the Aburra Valley of Antioquia province in northwestern Colombia (latitude: 6°09′52′′N, longitude: 75°25′23′′W), 1500 meters above the sea level. The mean temperature is 24°C.

### 2.2. Study Site

The study was conducted in the community of San Javier, which has a population of 135,885. In 2005 the incidence of DF in San Javier was 53/100,000 habitants—among the highest in Medellin (Secretaría de Salud de Medellin, personal communication, 22 February, 2007). San Javier is located in the central west area of Medellin, hemmed in by the steep hillside of the Aburra Valley. Three public medical clinics (La Quiebra [LQ], Villa Laura, and Santa Rosa de Lima) and one hospital with inpatient capability (San Javier Hospital (SJER)) serve the community. In addition there are private doctors providing care by appointment only, but the only private medical clinic providing acute care without appointment was El Divino Maestro (DM). This observational study was conducted in two public (LQ, SJER) and one private (DM) medical facilities. We assessed the patient populations of Villa Laura and Santa Rosa de Lima and found these clinics provided medical care by appointment only for nonacute and chronic medical issues.

### 2.3. Study Population

From March 2008 to March 2009, any resident of the study area regardless of age presenting to a participating medical facility with fever was eligible to participate. We defined fever as a measured temperature of ≥38.0°C using digital tympanic thermometers (that were provided for the study) at the time of presentation or history of fever in the preceding seven days. Patients were enrolled regardless of other presenting symptoms or signs, even if they suggested a focus for the infection. Informed consent was obtained from adults. For patients under 18 years of age, informed consent was obtained from the parent or guardian. To assure confidentiality, every patient received a study identification number upon enrollment. Only this number was included in subsequent forms. The study was approved by the ethical committee at the Instituto Colombiano de Medicina Tropical/University CES in Medellin and the Institutional Review Board of the International Vaccine Institute in Seoul, South Korea.

### 2.4. Study Design

As patients presented to participating clinics for medical care, study physicians reviewed chief complaints and vital signs to identify any patient with fever. Subjects were then consented and a full history and physical were completed by a study physician separate from the examination by the treating physician. The observations from the standardized history and physical were entered, real time, into a handheld computer by the study physician. The electronic case report forms on the hand-held devices were programmed with logic checks and legal values to ensure complete and accurate data. To ensure capture of all febrile subjects, the study coordinator also reviewed clinic records. The physician then collected an acute serum sample (3–5 mL) on each subject and scheduled a follow-up appointment 14–21 days later to collect a convalescent sample (3–5 mL). Specimens were transported daily to the diagnostic laboratory at the Instituto Colombiano de Medicina Tropical/Universidad CES for analysis.

### 2.5. Laboratory Tests

All acute samples were tested for dengue virus nucleic acids by reverse transcriptase-polymerase chain reaction (RT-PCR), NS1 antigen, and anti-dengue IgM and IgG antibodies. All convalescent samples were tested only for anti-dengue IgM antibodies.

Detection of dengue virus RNA and serotype identification was performed using the procedure described by Lanciotti et al. [5] and modified by Harris et al. [6]. Anti-dengue IgM was measured using the Dengue IgM Capture ELISA kit (Panbio, Brisbane, Australia). Viral NS1 antigen was detected either by Dengue Early ELISA (Panbio, Brisbane, Australia) or the Dengue NS1 Antigen ELISA test (Standard Diagnostics, Seoul, South Korea) based on availability of test kits. Anti-dengue IgG antibodies were detected using the IgG Indirect ELISA kit (Panbio, Brisbane, Australia). All these tests were performed according to the package inserts.

### 2.6. Case Definitions

We classified febrile events as follows. A laboratory-confirmed dengue case was a febrile patient with an acute serum specimen with detectable dengue viral RNA by RT-PCR, NS1 antigen, or IgM antibodies or a convalescent sample with detectable IgM antibodies. A dengue laboratory-negative case was a febrile patient with an acute specimen negative for dengue virus by RT-PCR, NS1, and IgM and also an IgM negative convalescent specimen. A dengue laboratory-indeterminate case was a febrile patient with an acute serum sample negative by RT-PCR, NS1 antigen, and IgM antibodies but no convalescent serum sample available for testing. We grouped dengue laboratory-negative and -indeterminate cases for analysis and called them other febrile illnesses (OFI). We further classified infections as primary or secondary based on whether or not a patient with a laboratory-confirmed dengue virus infection had detectable anti-dengue IgG antibodies in their acute serum specimen. Lastly we applied the 1997 WHO DF clinical case definitions for severity to the signs and symptoms reported by febrile patients—recently revised WHO criterion were not available at the time this study was designed [7].

### 2.7. Statistical Analysis

The Statistical Package for the Social Sciences (SPSS, version 15, Inc.01, Chicago, IL) was used for statistical analyses. Continuous data were described with medians and ranges and compared using the Mann-Whitney test. Nominal data were described by frequency and compared using the Chi-square test. We define statistical significance with a *P* value ≤ 0.05. There was no imputation of missing data; therefore, denominators vary by response.

## 3. Results

Between March 2008 and March 2009, 781 patients presented with a febrile illness out of a total of 12,327 persons seeking medical attention at the three participating health centers (LQ, DM, and SJER) in the San Javier community. Of them, 611 (78.2%) were treated as out-patients (ambulatory), and 170 (21.8%) were hospitalized. The ambulatory and hospitalized patients were similar with regard to age, sex, and ethnicity (Table 1).

Paired, acute and convalescent, serum specimens were obtained from 700 patients (89.6%); 566 (80.9%) were ambulatory patients and 134 (19.1%) were hospitalized. The remaining 81 (10.4%) had only acute specimens. A total of 73 patients (9.3%) had laboratory-confirmed dengue virus infections; 647 (82.8%) were laboratory-negative, and 61 (7.8%) were laboratory-indeterminate. The rate of positivity was similar among ambulatory and hospitalized patients, 9.2% (*n* = 56) versus 10.0% (*n* = 17), respectively. Twenty-six patients (3.3%) were positive by RT-PCR; 42 patients (5.4%) were positive by IgM antibodies; 4 patients (0.5%) were positive by Dengue Early ELISA (NS1); and another 3 (0.4%) were positive by Dengue NS1 Antigen ELISA. Two of the above patients were positive by both IgM and RT-PCR. Of the 26 RT-PCR positive, 20 of RT-PCR positive patients (76.9%) were DENV-2 positive; the remaining 6 (23.1%) were DENV-3 positive (Table 2). The presence of anti-dengue IgG antibodies in acute samples revealed 50 patients (68.5%) with laboratory-confirmed dengue virus infections were having their second or subsequent infections; however, IgG results are not available for 13 patients. Of note, the frequency of secondary infections was similar among ambulatory compared to hospitalized patients, 83.9% (47/56) versus 84.6% (11/13), respectively.

**Figure 1:**
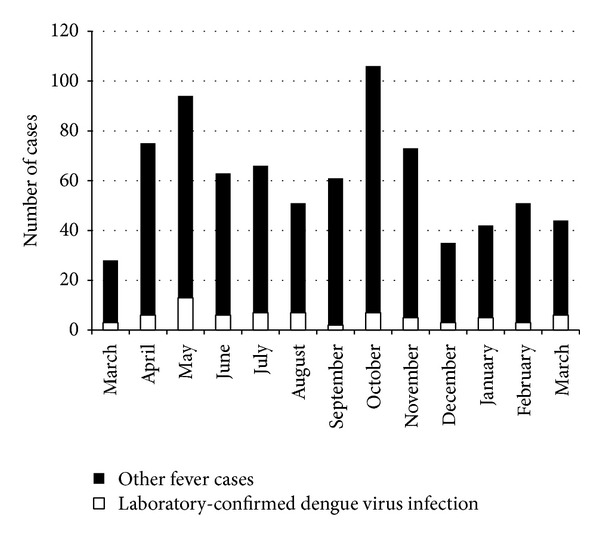
Number of febrile patients included in the study and dengue virus diagnostic results, by month, Medellin, Colombia, March 2008-2009 (*n* = 781).

Fever cases were identified in each month of the study; however the ratio of laboratory-confirmed dengue virus infections to OFI cases varied. The highest number of febrile patients occurred in October, but the highest number of laboratory-confirmed dengue virus infections, 13 (17.8%), occurred in May (Figure 1).

Compared to patients with OFI, patients with laboratory-confirmed dengue virus infections were statistically significantly younger (laboratory-confirmed dengue virus infections: median 5 years, range <1 to 63 versus OFI: median 11 years, range <1 to 85 years; *P* = 0.05) (Table 1). Although a slightly greater percentage of patients with laboratory-confirmed dengue virus infections were female and self-reported mestizos ethnicity (mixed European and Native American ancestry), these differences were not statistically significant.

Applying the WHO classification [1], all ambulatory and all but one hospitalized patient with laboratory-confirmed dengue virus infection met the clinical case definition of DF (fever and two or more of the following: retro-orbital pain, headache, rash, myalgia, arthralgia, leukopenia, or hemorrhagic manifestations). The remaining febrile patient did not meet criteria for DF.

The most frequently observed symptoms in patients with laboratory-confirmed dengue virus infections were anorexia (75.3% [*n* = 55]), asthenia (72.6% [*n* = 53]), and cough (67.1% [*n* = 49]) followed by headache (75.6% [*n* = 34]), vomiting (52.1% [*n* = 38]), rhinorrhea (49.3% [*n* = 36]), and nasal congestion (49.3% [*n* = 36]). Myalgias (53.3% [*n* = 24]), arthralgias (48.9% [*n* = 22]), and retro-orbital pain (44.4% [*n* = 20]) were less frequently observed. Rash was noted in 9.6% of patients (*n* = 7) with laboratory-confirmed dengue virus infections. Compared to patients with OFI, only neck pain was statistically significantly more common among patients with laboratory-confirmed dengue virus infections (51.1% [*n* = 23] versus 34.0% [*n* = 174]; *P* < 0.001).

Among the clinical signs, crepitus and rhonchi were statistically significantly more frequent in patients with laboratory-confirmed dengue virus infections than in those with OFI (20.5% [*n* = 15] versus 8.1% [*n* = 57], *P* < 0.001 for crepitus and 20.5% [*n* = 15] versus 11.7% [*n* = 83], *P* = 0.030 for rhonchi, resp.). In addition, the median pulse was statistically significantly higher (median for laboratory-confirmed dengue virus infections, 110 [range 58–180], versus OFI median, 100 [range 48–180], *P* = 0.026), and median diastolic blood pressure (median for laboratory-confirmed dengue virus infections, 60 [range 50–90], versus OFI median, 60 [range 50–110], *P* = 0.024) was statistically significantly lower in patients with laboratory-confirmed dengue virus infections than OFI patients (Table 3). There were no significant differences in the frequency of hemorrhagic manifestations, hematocrit, platelet counts, and white blood cell counts; however, complete blood counts were not available on all patients (Table 3).

In terms of clinical diagnosis, only 3 out of 73 (4.1%) patients with laboratory-confirmed dengue virus infections were diagnosed with dengue fever by the treating physician. One patient was ambulatory; the other two were hospitalized. The most common clinical diagnoses among patients with laboratory-confirmed dengue virus infections were viral syndrome (23.2% [*n* = 13]), tonsillitis/pharyngitis (17.9% [*n* = 14]), and acute diarrheal disease (8.9% [*n* = 5]) in ambulatory and pneumonia (35.3% [*n* = 6]) in hospitalized patients (Table 4). When the WHO clinical case definition for DF was applied to all febrile patients, the sensitivity was 98.6% and the specificity was 45.8%, giving a positive predictive value of 15.8% and the negative predictive value was 99.7% in this population.

## 4. Discussion

In the last two decades, Colombia and other Latin American countries have experienced marked increases in the incidence of both classic DF and DHF. Unfortunately, important aspects of dengue virus infection in the region, including clinical manifestations, age distribution, and disease burden, are unclear. In this study, we evaluated the frequency of dengue virus infection as well as its clinical presentation in ambulatory and hospitalized patients in several health facilities in the community of San Javier. Having tested all febrile patients regardless of presenting symptoms, the frequency of clinical signs and symptoms could also be compared between patients with laboratory-confirmed dengue virus infections and patients with OFI. The combination of different dengue specific tests provided us with a robust diagnostic panel that makes it unlikely that we have missed true dengue virus infections.

This one-year study showed that dengue virus infection is a significant medical concern in the San Javier community of Medellin. Dengue may be causing up to 9.3% of febrile illnesses in patients seeking medical attention. The frequency of dengue virus infections observed in the current study falls within the range reported in other Latin American countries. Previous studies have shown that incidence rates can vary significantly depending on sampling strategy (schoolchildren, community, and hospital cohorts), targeted population (pediatric versus adults), and the presence of an outbreak. The most comparable study was conducted by Ramos et al. in Patillas, Puerto Rico, in which all patients presenting to the only health center in the municipality with fever who met the WHO clinical DF case definition were tested regardless of the treating physician's diagnosis. In that study 11% were laboratory-positive [8]. In a cohort limited to school age children in Medellin (2010-2011), the annual incidence of laboratory-confirmed dengue virus infection was 11.1% when all febrile students absent from school were tested for dengue infection regardless of their chief complaint [9]. In Nicaragua during the 1998 DENV-3 epidemic, infection was confirmed in 60% (614/1027) of children with fever at the time of presentation, but testing was limited to subjects meeting the WHO DF case definition [10]. However, later in Nicaragua, a health center population-based study in 2002–2004 showed only 18% of children meeting the WHO case definition tested positive during a reintroduction of DENV-1 [11]. Similar positive rates were observed in Recife (Brazil) during 2004–2006; the study detected dengue virus in 54% (353/658) of patients ≥5 years of age and meeting the WHO DF case definition but also coincided with the reintroduction of DENV-3 in the country [12]. When the inclusion criterion for testing was expanded to include undifferentiated fever in Nicaraguan school children, on average 26% more laboratory-confirmed cases were identified each year [13]. Therefore, more symptomatic dengue infections were being identified among clinic patients even though the overall positivity rate was somewhat reduced (2001–2003 and 2004–2008 showed dengue infections rates between 5.8%–12%) [13]. Indeed, Lorenzi et al. confirmed at a tertiary hospital in Puerto Rico that as many as 11% of patients with undifferentiated fever requiring medical attention are laboratory-confirmed dengue virus infections that did not meet the WHO case definition [14]. Accepted case definitions, therefore, miss a significant number of the symptomatic dengue infections particularly if the patients are children and if the symptoms are not classical. Disease surveillance requires only a sampling of infections to detect changes that mark the beginning of outbreaks. Therefore, additional studies (e.g., cohort or population-based studies that test all fever cases, sequential serosurveys) are the most accurate way to understand the spectrum of dengue virus infection and disease burden.

Dengue cases were diagnosed every month of the year with higher frequency in May and October consistent with the typical nonoutbreak pattern in Colombia. This biphasic pattern corresponds to periods of increased rainfall. The same pattern is seen in Brazil but occurs during different months, February through May, indicating the importance of local weather patterns [15]. In addition, in Medellin it has been observed that the years with higher transmission of dengue were preceded by years with the climatic event “El Niño”; however, Suárez et al. did not find a statistical association with the climatic variables (rainfall, humidity, and temperature) [16].

The results of our study suggest that routine clinical laboratory tests could not differentiate patients with laboratory-confirmed dengue virus infection from those with OFI. The frequency of “typical” symptoms and clinical signs such as myalgias, arthralgias, retro-orbital pain, rash, and hemorrhages, including positive tourniquet test, was not significantly different; only neck pain, crepitus, and rhonchi, which are not typical DF symptoms, were more frequently observed in patients with laboratory-confirmed dengue virus infections. However, the clinical usefulness of this difference is questionable. Similarly, low counts of platelets, white blood cells, and neutrophils were not statistically associated with DF in these patients. Respiratory symptoms such as nasal congestion, rhinorrhea, and cough were frequently observed in both laboratory-confirmed dengue virus infections and OFI cases with no significant differences. This is different than findings in other studies and demonstrates the difficulty of clinical diagnosis [17–19].

A systematic review showed that the frequency of rash, myalgia, arthralgia, lethargy/prostration, and hemorrhagic signs was higher in patients with laboratory-confirmed dengue virus infections than patients with OFI, but adult patients accounted for most of these differences and only petechiae or positive tourniquet test was consistently higher in children with dengue [18]. A previous study in Colombia reported that rash and positive tourniquet test were more frequent in patients with DF than in other acute febrile syndromes [17]. In India the clinical manifestations more frequently observed were anorexia, myalgia, arthralgia, retro-orbital pain, and abdominal pain [20]. In Ratchaburi, Thailand, there was an association between gastrointestinal manifestations and severe dengue infection, but other findings were similar to those shown here; that is, there was not a great deal of specificity in clinical manifestations classically associated with symptomatic dengue virus infection [21]. Indeed, a study using surveillance data in Puerto Rico found no useful combination of signs or symptoms was sufficiently predictive among dengue virus laboratory-positive adults [22]. Lorenzi et al., in Puerto Rico, observed that 48.4% of the DF cases met the criteria for influenza-like illness of the Center for Disease Control: fever with cough or sore throat, although 79% influenza patients met WHO criteria for DF [14]. Taken together, treating physicians in dengue endemic areas need dengue diagnostics to identify dengue virus reliably because the presenting clinical picture is not sufficient to differentiate dengue virus infection from other febrile illness despite the fact that there is a significant amount of febrile disease caused by dengue virus.

This study may have been limited by the inability to distinguish through diagnostic tests true symptomatic dengue virus infections from coincidental asymptomatic infections. Several studies have confirmed that the rate of asymptomatic infection is at least the same or higher than the rate of symptomatic infection in the general population [23, 24]. However, all patients in this study were symptomatic at the time of testing making the probability of the infection being coincident with another infection low.

In conclusion, dengue, predominantly in its nonsevere form, proves to be an important cause of febrile illness in the Medellin area. The burden of disease is much larger than national surveillance data would suggest with roughly 10% of fevers regardless of the presenting symptoms are potentially caused by dengue infection. That said the clinical picture of the more mild infection we identified was nonspecific making it difficult to recognize but again accounting for the greatest burden of disease. Rapid, reliable, and inexpensive dengue diagnostics for use at point of care would greatly improve the accuracy of the medical diagnosis of the infection of dengue virus in the primary health care setting. However, a dengue vaccine would be more practical to prevent the disease and perhaps eliminate the diagnostic dilemma of this important cause of fever in endemic countries.

## Figures and Tables

**Table 1:** Characteristics of ambulatory and hospitalized fever patients, Medellin, Colombia, March 2008-2009.

Demographic characteristics	Ambulatory						Hospitalized						Total *n* = 781	
	DVI^a^		OFI^b^		Total *n* = 611		DVI		OFI		Total *n* = 170			
Sex, number (%)														
Males	19	(7.5)	234	(92.5)	253	(41.4)	10	(12.2)	72	(87.8)	82	(48.2)	335	(42.9)
Females	37	(10.3)	321	(89.7)	358	(58.6)	7	(8.0)	81	(92.0)	88	(51.8)	446	(57.1)
Race, number (%)														
Mestizos	49	(8.6)	519	(91.4)	568	(93.0)	16	(10.1)	143	(89.9)	159	(93.5)	727	(93.1)
Afrocolombian	7	(16.3)	36	(83.7)	43	(7.0)	1	(9.1)	10	(90.9)	11	(6.5)	54	(6.9)
Age														
Median age, yrs (range)	5	(0–63)	11	(0–85)	11	(0–85)	4	(0–48)	12	(0–85)	10	(0–85)	10	(0–85)
Others														
Median DPO^c ^(range)	2	(0–7)	2	(0–7)	2	(0–7)	2.5	(0–7)	2	(0–7)	2	(0–7)	2	(0–7)
Primary infection, number (%)	8	(17.0)	—	—	—	—	2	(15.4)	—	—	—	—	(10)	(16.7)
Secondary infection, number (%)^d^	39	(83.0)	—	—	—	—	11	(84.6)	—	—	—	—	(50)	(83.3)

^a^DVI: febrile, laboratory-confirmed dengue virus infection.

^b^OFI: other febrile illness.

^c^Days after onset of symptoms at presentation.

^d^13 patients are without IgG data: 9 ambulatory patients and 4 hospitalized patients.

**Table 2:** Dengue diagnostic results for ambulatory and hospitalized patients, Medellin, Colombia, March 2008-2009.

Test	Ambulatory			Hospitalized			Total		
	Positive	Tested	(%)	Positive	Tested	(%)	Positive	Tested	(%)
RT-PCR	17	611	2.8	9	170	5.3	26	781	3.3

Viral antigen (total)	6	602	1.0	1	166	0.6	7	768	0.9
SD NS1 antigen	3	602	0.5	0	166	0.0	3	768	0.4
Panbio (Early)	3	102	2.9	1	28	3.6	4	130	3.1

IgM (total)	34	611	5.6	8	170	4.7	42	781	5.4
Only one sample	4	45	8.9	1	29	3.4	5	74	6.8
Acute and convalescent positives	13	17	76.5	1	141	0.7	14	158	8.9
Seroconversion	17	566	3.0	6	140	4.3	23	706	3.3

Total	56	611	9.2	17	170	10.0	73	781	9.3

Two patients were laboratory positive by IgM and RT-PCR.

**Table 3:** Comparison of the clinical symptoms and signs of ambulatory and hospitalized patients with laboratory-confirmed dengue infections and other febrile illnesses. Medellin, Colombia, March 2008-2009.

Symptoms and signs			Ambulatory and hospitalized patients				
	All patients		DVI^a^		Other febrile illness cases		*P* value
	*N* = 781		*N* = 73		*N* = 708	
	Number	%	Number	%	Number	%
General status							
Asthenia	612	78.4	53	72.6	559	79.0	0.209
Head							
Headache^b^	457	82.0	34	75.6	423	82.6	0.236
Retro-orbital pain^b^	215	38.6	20	44.4	195	38.1	0.400
Neck pain^b^	197	35.4	23	51.1	174	34.0	**0.021**
Nasal congestion	412	52.8	36	49.3	376	53.1	0.536
Rhinorrhea	397	50.8	36	49.3	361	51.0	0.785
Sore throat	283	36.2	26	35.6	257	36.3	0.907
Flushed face	139	17.8	14	19.2	125	17.7	0.746
Injected conjunctiva	204	26.1	15	20.5	189	26.7	0.254
Red throat	333	42.6	27	37.0	306	43.2	0.305
Gastrointestinal							
Anorexia	585	74.9	55	75.3	530	74.9	0.927
Vomiting	365	46.7	38	52.1	327	46.2	0.338
Abdominal pain	307	39.3	27	37.0	280	39.5	0.669
Diarrhea	209	26.8	20	27.4	189	26.7	0.897
Respiratory							
Cough	531	68.0	49	67.1	482	68.1	0.867
Difficulty breathing	167	21.4	18	24.7	149	21.0	0.473
Crepitus	72	9.2	15	20.5	57	8.1	**<0.001**
Rhonchi	98	12.5	15	20.5	83	11.7	**0.030**
Pleural effusion	4	0.5	0	0.0	4	0.6	—
Other signs and symptoms							
Back pain^b^	245	44.0	19	42.2	226	44.1	0.874
Myalgias^b^	288	51.7	24	53.3	264	51.6	0.819
Arthralgias^b^	257	46.1	22	48.9	235	45.9	0.699
Rash	51	6.5	7	9.6	44	6.2	0.388
Hemorrhagic symptoms							
Positive tourniquet test^c^	28	3.6	2	2.7	26	3.7	0.938
Epistaxis	45	5.8	3	4.1	42	5.9	0.709
Bleeding gums	28	3.6	2	2.7	26	3.7	0.938
Hematemesis	12	1.5	1	1.4	11	1.6	0.705
Blood in stool	15	1.9	1	1.4	14	2.0	0.930
Blood in urine	11	1.4	1	1.4	10	1.4	0.622
Eccymoses	6	0.8	0	0.0	6	0.8	—
Petechiae	9	1.2	0	0.0	9	1.3	—
Physiological findings, median (range)							
Temperature (°C)	37.1	(35.3–40.1)	37.0	(35.6–40.0)	37.1	(35.3–40.1)	0.807
Systolic blood (mmHg)	100	(60–200)	99	(80–140)	100	(60–200)	0.147
Diastolic blood (mmHg)	60	(50–110)	60	(50–90)	60	(50–110)	0.024
Respiratory rate *x*/min	22	(14–100)	24	(16–100)	22	(14–80)	0.119
Pulse (beats/min)	100	(48–180)	110	(58–180)	100	(48–180)	0.026
Platelets (count/mm^3^×10^3^)^d^	376	(68–718)	332	(68–718)	363	(76–694)	0.771
Haematocrit (%)^d^	36.4	(21–55.4)	36.0	(21–47.3)	36.5	(22–55.4)	0.371
Neutrophils (%)^d^	70.6	(4.4–99.7)	63.3	(12.6–88.9)	71.5	(4.4–99.7)	0.135
White blood cell count^c^	12.5	(2.9–94)	14.3	(4.6–59)	12.4	(2.9–94)	0.360

^a^DVI: febrile, laboratory-confirmed dengue virus infection.

^b^The denominator all patients = 557, dengue laboratory positives = 45, and other febrile illness cases = 512 (patients ≥ 3 years old). To assure accurate reporting of pain symptoms, children under three years of age were excluded during the calculation of frequency of headache, retro-orbital pain, neck pain, myalgias, arthralgias, and joint pain.

^c^20 or more petechiae in 6.45 cm^2^.

^d^Complete for 17 dengue laboratory-positive cases and in 179 other febrile illness cases.

**Table 4:** Clinical diagnosis of laboratory-confirmed dengue fever cases and other febrile illness cases in ambulatory and hospitalized patients, Medellin, Colombia, March 2008-2009.

Diagnosis by physician	Ambulatory				Hospitalized			
	DF		OFI		DF		OFI	
	*n* = 56		*n* = 555		*n* = 17		*n* = 153	
	Number	%	Number	%	Number	%	Number	%
Dengue fever	1	1.8	36	5.9	2	11.8	4	2.6
Viral syndrome	13	23.2	80	13.1	0	0.0	9	5.8
Tonsillitis/pharyngitis	10	17.1	63	10.3	0	0.0	1	0.7
Diarrhea	5	8.9	38	6.2	0	0.0	8	5.2
Bronchitis	4	7.1	21	3.4	0	0.0	8	5.2
Otitis media	2	3.6	23	3.8	0	0.0	2	1.3
Pneumonia	1	1.8	19	3.1	6	35.3	30	19.6
Common cold/influenza	1	1.8	26	4.3	0	0.0	1	0.7
Urinary infection	0	0.0	34	5.6	1	5.9	24	15.7
Other	26	46.4	275	45.0	11	64.7	99	64.7

^a^DF: laboratory-confirmed dengue fever.

^b^OFI: other febrile illness.
